# Impact of elective minimally invasive sigmoidectomy for diverticular disease on quality of life: a single-center study

**DOI:** 10.1007/s13304-026-02812-6

**Published:** 2026-08-26

**Authors:** Daniele Sandonà, Giacomo Sarzo, Alvise Frasson, Emanuela Tessari, Daniele Gasparini, Cinzia Anna Maria Papappicco, Dario Gregori, Nicola Passuello

**Affiliations:** 1 Department of Surgical Oncological and Gastroenterological Sciences – DiSCOG, University of Padua, Padua, Italy; 2 OSA General Surgery, Padua University Hospital, University of Padua, Padua, Italy; 3 Unit of Biostatistics, Epidemiology and Public Health, Department of Cardiac, Thoracic, Vascular Sciences and Public Health, University of Padua, Padua,, Italy

**Keywords:** Diverticulitis, Sigmoidectomy, Quality of life, Patient-reported outcome measures, Minimally invasive surgery

## Abstract

The indication for elective sigmoidectomy after acute diverticulitis is shifting from prevention of complications to patient-centered outcomes. This study evaluated the impact of elective minimally invasive sigmoidectomy on quality of life (QoL) using a disease-specific tool and identified clinical factors associated with greater postoperative benefit. This was a retrospective analysis of a prospectively maintained database including patients who underwent elective minimally invasive sigmoidectomy for diverticular disease between June 2019 and February 2025. QoL was assessed preoperatively and six months postoperatively using the validated Diverticulitis Quality of Life (DvQoL) questionnaire. Primary outcomes includedthe change in DvQoL score (ΔDvQoL), the achievement of the minimal clinically important difference (MCID) and the patient acceptable symptom state (PASS). Ridge-penalized linear regression and Firth penalized logistic regression were used to identify predictors of QoL improvement. Ninety-two patients were analyzed(median age 63.5 years; 59.8% female). Median DvQoL scores significantly improved from 3.15 preoperatively to 0.55 at six months (*p* < 0.001). The MCID was achieved by 58.7% (n = 54), and the PASS was reached by 91.3% (n = 84). Multivariable analysis identified higher preoperative DvQoL score (*β* = 0.499, *p* < 0.001), older age (*p* = 0.042), and male sex (*p* = 0.018) as independent predictors of greater QoL improvement and MCID achievement. Surgical technique and disease severity (modified Hinchey classification) did not significantly influence QoL outcomes. Elective minimally invasive sigmoidectomy is associated witha significant and clinically meaningful short-term improvement in QoL, with preoperative symptom burden serving as the strongest predictor of benefit. However, due to the retrospective nature of the analysis and the lack of a conservative control group, these findings represent clinical associations rather than direct causality. The potential role of the DvQoL tool in shared decision-making and patient selection requires future validation in larger, multicenter prospective trials.

*Trial registration* Approved by the local Institutional Ethics Committee (Protocol number: 6585 N/AO/26).

## Introduction

Colonic diverticular disease is highly prevalent in Western countries, with incidence increasing with age and affecting more than half of individuals older than 80 years [1]. Although most patients remain asymptomatic, approximately 4% develop acute diverticulitis during their lifetime [2]. Recent data indicate a worldwide increase in the prevalence of colonic diverticulosis [3].

The clinical spectrum ranges from uncomplicated inflammatory episodes to complicated disease characterized by abscess, perforation, fistula, or obstruction [4]. Contrast-enhanced computed tomography represents the imaging modality of choice for diagnosis and staging, allowing accurate identification of complications and guiding management strategies [5]. Uncomplicated acute diverticulitis is usually treated conservatively, often in the outpatient setting, whereas complicated disease may require hospitalization, antibiotic therapy, percutaneous drainage of abscesses, or urgent surgical intervention in cases of generalized peritonitis or clinical deterioration [6]. Despite the current guidelines established by multiple scientific societies, significant variations exist in the surgical management of complicated diverticulitis in real-life clinical practice [7].

In the elective setting, the role of sigmoid colectomy after recovery from acute diverticulitis remains debated. Traditional recommendations favored elective resection after recurrent episodes to prevent further complications. However, contemporary guidelines advocate a more individualized approach, as the risk of severe recurrence is relatively low and the indication for surgery should not be based solely on the number of previous episodes. Instead, increasing emphasis has been placed on the impact of diverticular disease on patients’ quality of life [8–10].

Randomized trials such as the DIRECT and LASER studies have demonstrated that elective sigmoid resection may significantly improve quality of life in selected patients with recurrent or persistent symptoms, although this benefit must be balanced against the potential morbidity associated with surgery [11, 12]. To date, the results of the prospective multicenter study PREDIC-DIV, which aims to identify factors influencing quality of life after elective sigmoid resection for diverticular disease, are still awaited [13]. Consequently, identifying patients who are most likely to benefit from elective surgical treatment remains an important clinical challenge.

Disease-specific instruments have been developed to better quantify the burden of diverticular disease. The Diverticulitis Quality of Life (DvQoL) questionnaire is a validated tool designed to evaluate the impact of diverticulitis across physical, emotional, and behavioral domains, allowing clinically meaningful interpretation through parameters such as the minimal clinically important difference (MCID) and the patient acceptable symptom state (PASS) [14–16].

In this context, the present study aimed to evaluate the impact of elective sigmoid colectomy on quality of life in patients with diverticular disease using the DvQoL questionnaire and to identify clinical factors associated with greater postoperative benefit.

## Materials and methods

### Study design and population

Between June 2019 and February 2025, data from patients undergoing elective sigmoid colectomy for diverticular disease at our institution were prospectively collected and entered into a dedicated database.

Eligible patients were ≥ 18 years of age with a previous radiologic diagnosis of acute diverticulitis who were scheduled for elective surgical treatment. Patients undergoing emergency or urgent surgery and those with absolute surgical indications (e.g., fistula, bowel obstruction, or uncontrolled diverticular bleeding) were excluded. Patients unable to independently complete quality-of-life questionnaires were also excluded.

The study was conducted in accordance with the principles of the Declaration of Helsinki. The study protocol was reviewed by the local Institutional Ethics Committee (protocol number 6585N/AO/26), which acknowledged the observational nature of the study involving retrospective analysis of prospectively collected data. All patients provided written informed consent for the surgical procedure and for the use of their anonymized clinical data for research purposes. This observational study was conducted and reported in accordance with the STROBE (Strengthening the Reporting of Observational Studies in Epidemiology) guidelines [17].

### Preoperative assessment

All patients underwent standardized preoperative evaluation including medical history, physical examination, routine laboratory testing, and anesthesiology assessment. Operative risk was classified according to the American Society of Anesthesiologists (ASA) score, and comorbidities were quantified using the Charlson Comorbidity Index (CCI). An experienced radiologist reviewed the CT scans confirming the diagnosis of diverticulitis, and the radiological severity was recorded according to the modified Hinchey classification by Kaiser et al. [18]; when multiple CT scans were available, the one associated with the highest clinical severity was considered. Quality of life was assessed preoperatively using the DvQoL questionnaire. In our cohort, elective resection did not rely on strict protocols regarding episode frequency. Instead, a tailored approach based on shared decision-making was utilized. Indications were evaluated individually, considering the severity of past attacks, patient fitness regardless of advanced age, and informed patient preference after a thorough discussion of non-operative alternatives.

### Surgical procedure

All procedures were performed electively using a minimally invasive approach (laparoscopic or robotic) at a high-volume colorectal center by surgeons with different levels of experience, following a standardized institutional technique.

The operation consisted of sigmoid resection with restoration of bowel continuity through stapled colorectal anastomosis. Dissection was performed using a medial-to-lateral approach with identification and preservation of the ureter and gonadal vessels. The inferior mesenteric artery was divided either at its origin or distally according to surgeon preference. Proximal resection was carried out in macroscopically healthy colon, while distal transection was performed at the level of the upper rectum. Splenic flexure mobilization was performed when necessary to obtain a tension-free anastomosis. Intraoperative perfusion assessment using indocyanine green fluorescence was routinely performed prior to colonic transection. Recorded intraoperative variables included surgical approach, primary surgeon experience, operative time, splenic flexure mobilization, level of inferior mesenteric artery ligation, estimated blood loss, and postoperative intensive care unit admission.

### Postoperative management and follow-up

Perioperative care followed an Enhanced Recovery After Surgery (ERAS) protocol. Collected postoperative data included time to oral intake, time to first flatus and bowel movement, postoperative C-reactive protein levels on day 3, length of hospital stay, and pathologic specimen characteristics. Thirty-day postoperative complications were recorded and graded according to the Clavien–Dindo classification. Clinical follow-up was conducted at six months after surgery and included repeat administration of the DvQoL questionnaire.

### Quality of life assessment

Quality of life was assessed using the validated DvQoL questionnaire administered preoperatively and six months after surgery. The questionnaire consists of 17 items scored on a Likert scale and evaluates the impact of diverticular disease across physical, emotional, and behavioral domains. DvQoL scores were calculated according to published recommendations and transformed to a 0–10 scale, with higher scores indicating worse quality of life [14]. Specifically, we utilized the version validated for the Italian population to ensure accurate and culturally adapted measurements [16]. The minimal clinically important difference (MCID) was defined as an improvement of ≥ 2.2 points, while the patient acceptable symptom state (PASS) threshold was defined as a postoperative score < 3.2 [15]. Clinical improvement was defined as the difference between preoperative and postoperative scores, with positive values indicating symptom reduction. Patients were classified according to achievement of MCID and PASS.

### Statistical analysis

Normally distributed variables are reported as mean ± standard deviation (SD), whereas non-normally distributed variables are presented as median and interquartile range (IQR). Categorical variables are expressed as absolute frequencies and percentages. Postoperative complications were reported descriptively and classified according to the Clavien–Dindo classification.

The change in quality of life was calculated as the difference between baseline and 6-month postoperative DvQoL scores (ΔDvQoL = preoperative − postoperative score). Pre vs post comparison was performed using the Wilcoxon signed-rank test. Effect size was quantified using rank-biserial correlation.

Associations between ΔDvQoL and clinical variables were initially explored using a Spearman correlation matrix. Variables considered clinically relevant were then included in a multivariable linear regression model with Ridge penalization to identify factors independently associated with changes in DvQoL. Model assumptions were evaluated through residual diagnostics, assessment of homoscedasticity, and evaluation of multicollinearity using variance inflation factors (VIF).

Achievement of the MCID was analyzed as a binary outcome. Associations between clinically relevant predictors and MCID attainment were evaluated using Firth’s penalized logistic regression, which reduces small-sample bias in maximum likelihood estimation and provides stable estimates in the presence of potential separation. Odds ratios (ORs) with profile likelihood 95% confidence intervals were reported [19].

In addition, a Ridge-penalized logistic regression model was fitted to explore the predictive performance of the model. Ridge penalization shrinks regression coefficients toward zero, thereby reducing overfitting and improving model stability in small datasets. The optimal penalty parameter (λ) was determined by maximizing the corrected Akaike Information Criterion (AICc). Model discrimination was assessed using the area under the receiver operating characteristic curve (AUC). Internal validation of the penalized models was performed using bootstrap optimism correction with 300 resamples according to Harrell’s methodology. [20, 21]

All statistical analyses were performed using R software (R Foundation for Statistical Computing, Vienna, Austria) [22]. A two-sided *p* value < 0.05 was considered statistically significant.

## Results

### Patient characteristics

A total of 92 patients undergoing elective sigmoid colectomy for diverticular disease were included in the study. Baseline demographic and clinical characteristics are reported in Table 1. Median age was 64 years (IQR 55–71). with a female predominance (n = 55, 60% vs. n = 37, 40% males). The median body mass index (BMI) was 24.9 kg/m^2^ (IQR 22.8–26.9 kg/m^2^). Regarding smoking status, the majority of patients were non-smokers (n = 64, 70%), while 19 patients (21%) were current smokers and 9 (9.8%) were former smokers. According to the modified Hinchey classification, the majority of the population presented with milder or localized forms of diverticulitis: 27 patients (29.3%) presented with mild clinical diverticulitis (Stage 0), 42 patients (45.7%) had a confined pericolic inflammation or phlegmon (Stage 1a), and 20 patients (21.7%) had a pericolic or mesocolic abscess (Stage 1b). More severe presentations were less frequent, with pelvic or distant abscesses (Stage 2) observed in 1 patient (1.1%) and generalized purulent peritonitis (Stage 3) reported in 2 patients (2.2%). No cases of generalized fecal peritonitis (Stage 4) were recorded in this series. Regarding the surgical management, the two patients presenting with generalized purulent peritonitis (Hinchey Stage 3) were managed with non-operative management (NOM) during the acute phase. Following the successful resolution of the acute episode, these patients, along with the rest of the cohort, underwent elective surgical treatment, which consisted of laparoscopic resection and primary anastomosis; conversion to open surgery was necessary for 3 patients (3.3%).

**Table 1:** Baseline characteristics of the study population

Variable	N = 92^1^
Age	64 (55, 71)
Unknown	0
BMI	24.9 (22.8, 26.9)
Unknown	0
*Smoke*	
Never	64 (70%)
Current	19 (21%)
Previously	9 (9.8%)
Unknown	0
*Gender*	
Male	37 (40%)
Female	55 (60%)
Other	0 (0%)
Unknown	0
*Modified Hinchey*	
0—Mild clinical diverticulitis	27 (29.3%)
1a—Confined pericolic inflammation or phlegmon	42 (45.7%)
1b—Pericolic or mesocolic abscess	20 (21.7%)
2—Pelvic, distant, intra-abdominal or retroperitoneal abscess	1 (1.1%)
3—Generalized purulent peritonitis	2 (2.2%)
4—Generalized fecal peritonitis	0 (0%)
^1^ Median (Q1, Q3); n (%)	

### Postoperative outcomes

Thirty-day postoperative complications occurred in 22.8% of patients and are summarized in Table 2 according to the Clavien–Dindo classification. Major complications (≥ grade III) occurred in 3.3% of cases (one grade IIIa and two grade IIIb). No postoperative mortality was observed. Specifically, the anastomotic leak rate was 6.5% (6 patients). Re-interventions were required in 3 cases (3.3%). The median postoperative length of stay (LOS) was 6 days (IQR 3–8). No postoperative mortality was observed.

**Table 2:** Postoperative complications according to the Clavien-Dindo classification

Clavien Dindo classification	N = 92*1*
Grade 0: No complications	71 (77.2%)
Grade I: Any minor deviation from the normal course without intervention	8 (8.7%)
Grade II: Requiring pharmacological treatment	10 (10.9%)
Grade IIIa: Intervention without general anesthesia	1 (1.1%)
Grade IIIb: Intervention under general anesthesia	2 (2.2%)
Grade IVa: Single-organ dysfunction	0 (0%)
Grade IVb: Multiorgan dysfunction	0 (0%)
Grade V: Death of the patient	0 (0%)
*1* n (%)	

### Quality of life

**Fig. 1:**
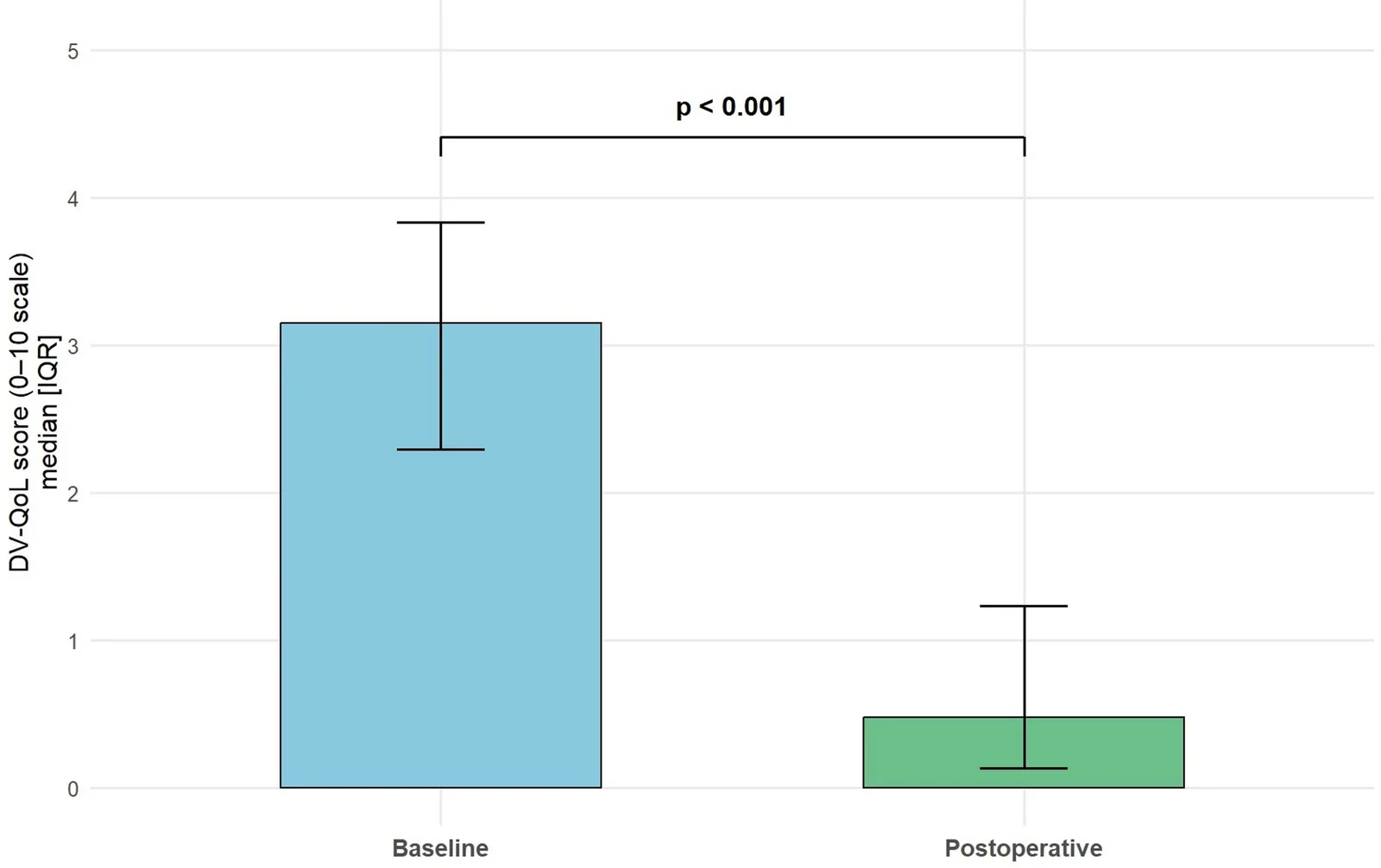
Preoperative versus 6-month postoperative diverticulitis quality of life (DvQoL) scores

**Fig. 2:**
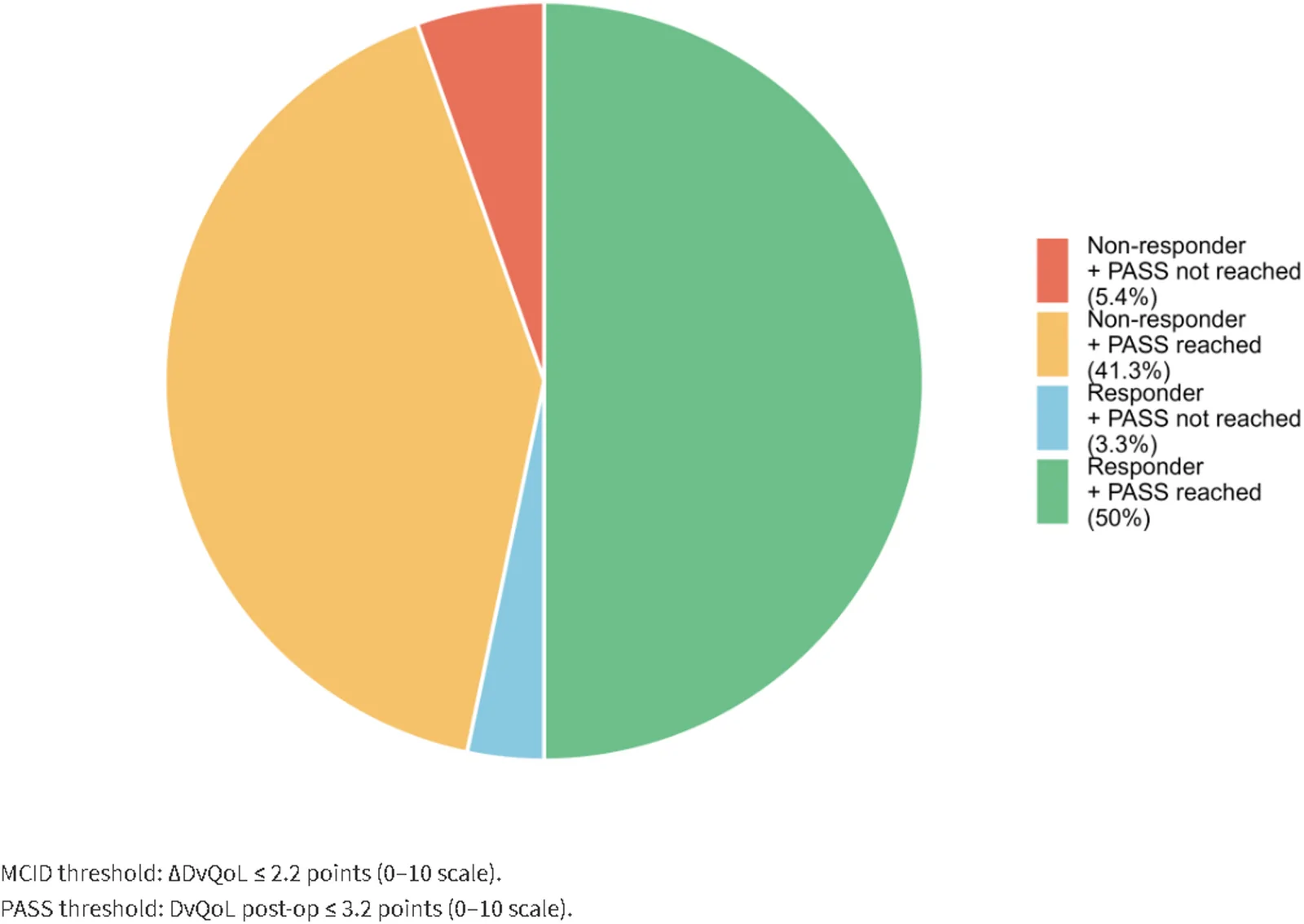
MCID and PASS attainment at follow up

The median DvQoL score improved from 3.151 (IQR 1.507) preoperatively to 0.548 (IQR 1.096) six months after surgery. The mean change in DvQoL (ΔDvQoL) was 2.33 ± 1.29, corresponding to a statistically significant improvement in quality of life as confirmed by the Wilcoxon signed-rank test (*p* < 0.001) with a large effect size (rank-biserial correlation = 1) (Fig. 1). Overall, 54 patients (58.7%) achieved the MCID (responder), while 84 patients (91.3%) reached the PASS. Five patients (5.4%) achieved neither MCID nor PASS, although none experienced a worsening in quality of life (ΔDvQoL ≥ 0) (Fig. 2).

### Correlations and multivariable analysis

Spearman correlation analysis was performed to explore associations between ΔDvQoL and clinical variables. Among the variables examined, the baseline DvQoL score showed the strongest correlation with ΔDvQoL (*ρ* = 0.64, *p* < 0.05). No significant correlations were observed between ΔDvQoL and other demographic, clinical, or intraoperative variables, including age, BMI, operative time, blood loss, ASA score, smoking status, modified Hinchey classification, surgical technique, and surgeon experience (all *p* > 0.05).

In the Ridge multivariable linear regression model (Table 3), the preoperative DvQoL score emerged as the independent factor most strongly associated with improvement in quality of life (*β* = 0.499; SE = 0.064; *p* < 0.001). Age (*p* = 0.042) and sex (*p* = 0.018) were also significantly associated with quality-of-life improvement. The model explained 44,2% of the variance (adjusted R^2^ = 0.442). No evidence of multicollinearity was detected (variance inflation factor values close to 1). Internal validation was performed using Harrell's Bootstrap with 300 resamples. No significant differences in ΔDvQoL were observed according to ASA class or modified Hinchey stage.

**Table 3:** Multivariable Ridge linear regression of ΔDvQoL

Term	*β* (Ridge)	95% CI low	95% CI high	*p* value
Age (years)	0.020	0.001	0.038	0.042
Gender (Female vs Male)	−0.486	−0.879	−0.092	0.017
DvQoL pre op (0–10)	0.499	0.374	0.625	< 0.001
ASA (2 vs 1)	0.145	−0.355	0.644	0.572
ASA (3 vs 1)	−0.076	−0.610	0.458	0.781
Hinchey (1a vs 0)	0.226	−0.193	0.644	0.293
Hinchey (1b vs 0)	0.059	−0.418	0.536	0.808
Hinchey (2 vs 0)	0.198	−0.547	0.943	0.603
Hinchey (3 vs 0)	0.178	−0.531	0.886	0.624

Clinically relevant variables were included in a Firth penalized logistic regression model to identify factors associated with MCID achievement (Table 4). Preoperative DvQoL score, age and male sex were all significantly associated with MCID achievement (*p* < 0.05). The model showed satisfactory discrimination with an apparent area under the ROC curve (AUC) of 0.841. The AUC is reported for descriptive purposes and not as a measure of clinically applicable predictive performance. No multicollinearity was observed (VIF values close to 1).

**Table 4:** Results of a Firth penalized logistic regression model

Predictor	OR	95% CI low	95% CI high	*p* value
Age (years)	1.05	1	1.12	0.045
Gender (Female vs Male)	0.26	0.07	0.85	0.025
DvQoL pre op (0–10)	11.09	3.51	50.2	< 0.001
ASA (2 vs 1)	1.3	0.3	5.74	0.723
ASA (3 vs 1)	1.43	0.29	7.02	0.656
Hinchey (1a vs 0)	1.78	0.55	5.89	0.338
Hinchey (1b vs 0)	1.24	0.31	5.04	0.755
Hinchey (2 vs 0)	0.65	0.02	112.8	0.820
Hinchey (3 vs 0)	6.81	0.34	162.86	0.199

An exploratory prediction model based on Ridge regression was developed to estimate the probability of achieving the Minimal Clinically Important Difference (Table 5). The predictor contributing most to the model is preoperative DvQoL score. The model showed good discrimination, with an empirical AUC of 0,84 and a corrected AUC of 0.79, and calibration, with a corrected Brier Score of 0.19 and a non-significant Hosmer–Lemeshow Test (*p* = 0.38). Calibration plots confirmed good agreement between predicted and observed outcomes. Model interpretability analyses using partial dependence plots and SHAP values identified the variables contributing most to the prediction. Internal validation based on bootstrap resampling (300 repetitions) demonstrated limited optimism and acceptable internal stability.

**Table 5:** Multivariable Ridge logistic regression for prediction of MCID achievement

Predictor	OR	95% CI low	95% CI high	p value
Age (years)	1.04	0.99	1.08	0.115
Gender (Female vs Male)	0.4	0.15	1.08	0.070
DvQoL pre op (0–10)	6.18	2.57	14.89	< 0.001
ASA (2 vs 1)	1.42	0.43	4.7	0.567
ASA (3 vs 1)	1.46	0.41	5.18	0.558
Hinchey (1a vs 0)	1.59	0.58	4.42	0.370
Hinchey (1b vs 0)	1.12	0.36	3.54	0.844
Hinchey (2 vs 0)	1.5	0.25	9.03	0.659
Hinchey (3 vs 0)	1.67	0.31	9	0.549

## Discussion

The present study demonstrates that elective sigmoid colectomy for diverticular disease is associated with a significant and clinically meaningful improvement in quality of life as assessed by the disease-specific DvQoL questionnaire. The marked reduction in DvQoL scores observed six months after surgery, together with the large effect size, indicates that the benefit of surgical treatment is not only statistically significant but also clinically relevant from the patient’s perspective. However, given the observational design and the absence of a non-operative control group, these findings should be interpreted as associative rather than causal.

These findings are consistent with the results of randomized trials such as the DIRECT and LASER trials, which demonstrated improved quality of life in patients undergoing elective sigmoid resection compared with conservative management. An important aspect of the present study is the use of the DvQoL questionnaire, a disease-specific instrument with validated thresholds for MCID and PASS. In contrast to many previous studies that relied on generic patient-reported outcome measures such as the Gastrointestinal Quality of Life Index (GIQLI), the Short Form-36 (SF-36), or the EuroQol-5 Dimensions (EQ-5D), the use of a disease-specific tool allows a clearer distinction between numerical score changes and clinically meaningful patient-perceived benefit. Furthermore, the prospective collection of data reduces potential biases related to retrospective study designs, although it does not eliminate selection bias inherent to surgical indication.

In our cohort, 58.7% of patients achieved the MCID, while more than 90% reached the PASS threshold. This finding suggests that even relatively modest quantitative improvements may translate into a clinically satisfactory symptom state, particularly in patients with less severe baseline symptoms. These results support the concept that quality of life outcomes should be interpreted not only in terms of score variation but also in relation to patient-centered thresholds of clinical relevance.

The analysis of factors associated with postoperative improvement showed that the preoperative DvQoL score was the strongest determinant of quality-of-life change. This observation is clinically plausible and suggests that patients with more impaired baseline quality of life have greater potential to benefit from surgical intervention. However, this association may be partially driven by statistical phenomena such as regression to the mean and ceiling effects, and therefore should not be interpreted as a purely causal relationship. In this perspective, the preoperative DvQoL score may represent a useful tool within the shared decision-making process, potentially contributing to a more individualized selection of surgical candidates, although its predictive role requires external validation before clinical implementation.

In addition to baseline symptom burden, demographic factors such as age and sex were independently associated with quality-of-life outcomes. In the multivariable linear regression model, male sex was associated with a greater mean improvement in DvQoL score compared with female sex, while increasing age was also positively associated with the magnitude of improvement. These findings were consistent in the analysis of the dichotomous outcome represented by MCID achievement using Firth penalized logistic regression. A similar association between male sex and favorable outcomes has been reported in previous studies, including the analysis by Posabella et al. [23]. This disparity warrants a deeper interpretation of gender-based disease experience. In literature, female patients often report more persistent functional symptoms after an episode of diverticulitis, frequently overlapping with an Irritable Bowel Syndrome (IBS) pattern [24]. However, this interpretation remains speculative, as IBS-related symptoms and psychosocial factors were not directly assessed in the present study. These IBS-like symptoms may be less responsive to the anatomical correction provided by surgery compared to the primary inflammatory symptoms. Future studies incorporating functional and psychological assessments are needed to clarify this aspect. It remains an open question whether the Dv-QoL questionnaire fully captures these functional nuances or if there is an underlying bias in pain perception and reporting between genders. Our data suggest that while surgery is effective for both, the ceiling for symptomatic relief might be lower in women due to these associated functional overlays.

The observation that male and older patients experienced greater improvements should not necessarily be interpreted as evidence of greater intrinsic effectiveness of surgery in these subgroups. Differences in symptom perception, preoperative expectations, or disease experience may partly explain these findings. In this context, Dv-QoL outcomes should primarily be interpreted as a reflection of patient-reported experience rather than purely objective clinical parameters. Historically, there has been a prejudice against elective surgery in the elderly, often reserved only for acute complications due to perceived frailty. However, our results indicate that older patients stand to gain the most in terms of quality of life. This finding should be interpreted cautiously, as it may be influenced by selection bias, with fitter elderly patients more likely to be offered surgery. The benefit-to-risk ratio in the elective setting appears favorable even in advanced age, although this conclusion requires confirmation in larger and more representative populations.

Conversely, several clinical and intraoperative variables commonly considered relevant (including modified Hinchey classification, ASA score, surgical approach, and surgeon experience) were not significantly associated with quality-of-life improvement in our experience. This lack of association may be related to limited statistical power and should not be interpreted as evidence of absence of effect. This suggests that, in the elective setting, patient-perceived benefit may be driven predominantly by preoperative symptom burden rather than by anatomical disease severity or technical aspects of the procedure, although this hypothesis warrants further investigation.

Regarding procedural safety, the postoperative complication rate observed in this study (22.8%), with a major complication rate of 3.3% and no mortality, is consistent with previously reported outcomes for elective colorectal surgery [25]. These findings support the safety of elective sigmoid resection when performed in an appropriate elective setting and in experienced centers. However, the potential impact of postoperative complications on patient-reported quality of life was not specifically analyzed and represents an important area for future research.

Several limitations should be acknowledged. First, the relatively small sample size limits the statistical power of the analyses and supports an exploratory and associative interpretation of the results. Second, the observational design and the absence of an external validation cohort limit the clinical applicability of the exploratory prediction model. The analytical strategy adopted—using Firth penalized logistic regression to reduce small-sample bias and Ridge regression for exploratory modeling—allowed a more robust estimation of associations but does not overcome the intrinsic limitations of the study design. Third, the absence of a conservatively treated control group prevents causal inference regarding the comparative effectiveness of surgical versus non-surgical management. Finally, the follow-up was limited to six months. While long-term data are always desirable, six months represents a critical physiological and psychological crossroads: it is a sufficient timeframe for the resolution of post-surgical inflammation and the stabilization of new bowel habits, yet it is short enough to minimize recall bias or the interference of unrelated life events on quality-of-life scores.

Overall, these findings should therefore be interpreted primarily as hypothesis-generating and may provide a basis for future prospective multicentric studies aimed at developing validated predictive models for patient selection.

## Conclusions

Elective sigmoid colectomy for diverticular disease is associated with a significant improvement in patient-reported quality of life at six months after surgery. The preoperative Dv-QoL score appears to be the main determinant of postoperative improvement, while demographic factors such as age and sex may also influence outcomes.

These findings suggest that disease-specific quality-of-life assessment may play a valuable role in the selection of candidates for elective surgery and in the shared decision-making process. Larger prospective and multicenter studies with external validation are required to confirm these associations and to translate them into clinically applicable decision-support tools.

## Data Availability

The datasets generated and analyzed during the current study are available from the corresponding author on reasonable request.

## References

[1] Stollman N, Raskin JB (2004) Diverticular disease of the colon. Lancet 363(9409):631–639. 10.1016/S0140-6736(04)15597-9 14987890

[2] Shahedi K, Fuller G, Bolus R, Cohen E, Vu M, Shah R et al (2013) Long-term risk of acute diverticulitis among patients with incidental diverticulosis found during colonoscopy. Clin Gastroenterol Hepatol 11(12):1609–1613. 10.1016/j.cgh.2013.06.020 PMC5731451 23856358

[3] Violi A, Cambiè G, Miraglia C, Barchi A, Nouvenne A, Capasso M et al (2018) Epidemiology and risk factors for diverticular disease. Acta Biomed 89(9-S):107–112. 10.23750/abm.v89i9-S.7924 PMC6502189 30561403

[4] Andersen JC, Bundgaard L, Elbrønd H, Laurberg S, Walker LR, Støvring J; Danish Surgical society. Danish national guidelines for treatment of diverticular disease. Dan Med J. 2012 May;59(5):C4453. 22549495

[5] Tiralongo F, Di Pietro S, Milazzo D, Galioto S, Castiglione DG, Ini’ C et al (2023) Acute colonic diverticulitis: CT findings, classifications, and a proposal of a structured reporting template. Diagnostics (Basel) 13(24):3628. 10.3390/diagnostics13243628 PMC10742435 38132212

[6] Francis NK, Sylla P, Abou-Khalil M, Arolfo S, Berler D, Curtis NJ et al (2018) EAES and SAGES 2018 consensus conference on acute diverticulitis management: evidence-based recommendations for clinical practice. Surg Endosc 33(9):2726–2741. 10.1007/s00464-019-06882-z PMC6684540 31250244

[7] Huo B, Massey LH, Seitidis G, Mavridis D, Antoniou SA, EAES Diverticulitis Survey Advisory Group (2025) Variation in the surgical management of complicated diverticulitis: a cross-sectional study of European surgeons. Surg Endosc 39(2):691–698. 10.1007/s00464-024-11456-9 39719489

[8] Schultz JK, Azhar N, Binda GA, Barbara G, Biondo S, Boermeester MA et al (2020) European society of coloproctology: guidelines for the management of diverticular disease of the colon. Colorectal Dis 22(Suppl 2):5–28. 10.1111/codi.15140 32638537

[9] Sartelli M, Weber DG, Kluger Y, Ansaloni L, Coccolini F, Abu-Zidan F et al (2020) 2020 update of the WSES guidelines for the management of acute colonic diverticulitis in the emergency setting. World J Emerg Surg 15(1):32. 10.1186/s13017-020-00313-4 PMC7206757 32381121

[10] Fugazzola P, Ceresoli M, Coccolini F, Gabrielli F, Puzziello A, Monzani F et al (2022) The WSES/SICG/ACOI/SICUT/AcEMC/SIFIPAC guidelines for diagnosis and treatment of acute left colonic diverticulitis in the elderly. World J Emerg Surg 17(1):5. 10.1186/s13017-022-00408-0 PMC8781436 35063008

[11] Van de Wall BJM, Stam MAW, Draaisma WA, Stellato R, Bemelman WA, Boermeester MA et al (2017) Surgery versus conservative management for recurrent and ongoing left-sided diverticulitis (DIRECT trial): an open-label, multicentre, randomised controlled trial. Lancet Gastroenterol Hepatol 2(1):13–22. 10.1016/S2468-1253(16)30109-1 28404008

[12] Santos A, Mentula P, Pinta T, Ismail S, Rautio T, Juusela R et al (2025) Sigmoid resection vs conservative treatment after diverticulitis: prespecified 4-year analysis of the LASER randomized clinical trial. JAMA Surg 160(6):615–622. 10.1001/jamasurg.2025.0572 PMC11983291 40202724

[13] Sohn M, Agha A, Iesalnieks I, Bremer S, Trum S, Di Cerbo F et al (2020) PREDICtors for health-related quality of life after elective sigmoidectomy for DIVerticular disease: the PREDIC-DIV study protocol of a prospective multicentric transnational observational study. BMJ Open 10(3):e034385. 10.1136/bmjopen-2019-034385 PMC7202696 32209628

[14] Spiegel BM, Reid MW, Bolus R, Whitman CB, Talley J, Dea S et al (2015) Development and validation of a disease-targeted quality of life instrument for chronic diverticular disease: the DV-QOL. Qual Life Res 24(1):163–179. 10.1007/s11136-014-0753-1 25059533

[15] Khor S, Flum DR, Strate LL, Hantouli MN, Harris HM, Lavallee DC et al (2021) Establishing clinically significant patient-reported outcomes for diverticular disease. J Surg Res 264:20–29. 10.1016/j.jss.2021.01.045 PMC9011199 33744774

[16] Polese L, Bressan A, Savarino E, Vecchiato M, Turoldo A, Frigo A et al (2018) Quality of life after laparoscopic sigmoid resection for uncomplicated diverticular disease. Int J Colorectal Dis 33(5):513–523. 10.1007/s00384-018-3005-y 29525902

[17] Cuschieri S (2019) The STROBE guidelines. Saudi J Anaesth 13(Suppl 1):S31–S34. 10.4103/sja.SJA_543_18 PMC6398292 30930717

[18] Kaiser AM, Jiang JK, Lake JP, Ault G, Artinyan A, Gonzalez-Ruiz C, Essani R, Beart RW Jr. (2005) The management of complicated diverticulitis and the role of computed tomography. Am J Gastroenterol 100(4):910–917. 10.1111/j.1572-0241.2005.41154.x 15784040

[19] Wang X (2014) Firth logistic regression for rare variant association tests. Front Genet 5:187. 10.3389/fgene.2014.00187 PMC4063169 24995013

[20] Ambler G, Seaman S, Omar RZ (2012) An evaluation of penalised survival methods for developing prognostic models with rare events. Stat Med 31(11–12):1150–1161. 10.1002/sim.4371 21997569

[21] Harrell FE Jr, Lee KL, Mark DB (1996) Multivariable prognostic models: issues in developing models, evaluating assumptions and adequacy, and measuring and reducing errors. Stat Med 15(4):361–387. 10.1002/(SICI)1097-0258(19960229)15:4<361::AID-SIM168>3.0.CO;2-4 8668867

[22] R Core Team. R: A language and environment for statistical computing (Version 4.5.2). Vienna, Austria: R Foundation for Statistical Computing; 2025. Available from: https://www.R-project.org/

[23] Posabella A, Steinemann DC, Droeser RA, Varathan N, Ayçiçek SG, Nocera F et al (2021) Predictive model estimating the decrease of postoperative gastrointestinal quality of life index (GIQLI) in patients after elective laparoscopic sigmoid resection for diverticular disease. Langenbecks Arch Surg 406(5):1571–1580. 10.1007/s00423-021-02186-w PMC8370950 34031729

[24] Alamo RZ, Quigley EMM (2019) Irritable bowel syndrome and colonic diverticular disease: overlapping symptoms and overlapping therapeutic approaches. Curr Opin Gastroenterol 35(1):27–33. 10.1097/MOG.0000000000000499 30407258

[25] Nocera F, Haak F, Posabella A, Angehrn FV, Peterli R, Müller-Stich BP et al (2023) Surgical outcomes in elective sigmoid resection for diverticulitis stratified according to indication: a propensity-score matched cohort study with 903 patients. Langenbecks Arch Surg 408(1):295. 10.1007/s00423-023-03034-9 PMC10400669 37535118

